# Third molar eruption in orthopantomograms as a feature for forensic age assessment—a comparison study of different classification systems

**DOI:** 10.1007/s00414-023-02982-7

**Published:** 2023-03-08

**Authors:** Maximilian Timme, Jan Viktorov, Laurin Steffens, Adam Streeter, André Karch, Andreas Schmeling

**Affiliations:** 1 Institute of Legal Medicine, University Hospital Muenster, Röntgenstraße 23, 48149 Münster, Germany; 2 Institute of Epidemiology and Social Medicine, University of Muenster, Domagkstraße 3, 48149 Münster, Germany

**Keywords:** Age assessment, Dental age, Eruption, Third molar, Orthopantomogram

## Abstract

Evaluation of third molar eruption represents an established method for age assessment of living individuals. Different classification systems are available for the radiological assessment of third molar eruption. The aim of this study was to identify the most accurate and reliable classification system for the mandibular third molar eruption on orthopantomograms (OPG). We compared the method of Olze et al. (2012) with the method of Willmot et al. (2018) and a newly derived classification system using OPGs from 211 individuals aged 15–25 years. The assessments were performed by three experienced examiners. One examiner evaluated all radiographs twice. The correlation between age and stage was investigated and the inter- and intra-rater reliability was estimated for all three methods. Correlation between stage and age was similar between classification systems, although higher in the data from males (Spearman’s rho ranging from 0.568 to 0.583) than from females (0.440 to 0.446). Inter- and intra-rater reliability measures were similar across methods and invariant on sex, with overlapping confidence intervals, although the highest point estimates for both intra- and inter-rater reliability were for the method by Olze et al. with Krippendorf’s alpha values of 0.904 (95% confidence interval 0.854, 0.954) and 0.797 (95% confidence interval 0.744, 0.850). It was concluded that the method of Olze et al. from 2012 is a reliable method for practical application and future studies.

## Introduction

Forensic age assessment can be consulted whenever a person’s age is unknown or when there is reasonable doubt about a person’s age. Thus, forensic age assessment has a particular role in the context of legal proceedings or administrative acts [1–4]. It involves determining the developmental status of various feature systems and comparing them with reference populations [5]. In this way, with a known margin of inaccuracy, the age of the person under evaluation can be estimated. Following the recommendations of the study group on forensic age diagnostics (AGFAD) for age assessment in living individuals, various methods should be combined, and a dental examination should always be part of the procedure [2, 3, 5].

A dental feature suitable for age assessment is the development of the third molars [6–10]. Mineralization and eruption into the oral cavity can be evaluated on the third molars. It has long been known that eruption, initially assessed clinically only, is a feature suitable for age assessment [11]. Some literature, for example, reports that as early as ancient Rome, the age for military service of young men was determined by the eruption of certain teeth into the oral cavity [12]. However, modern reports on the evaluation of tooth eruption for age assessment also go back a long way. In 1837, a work was published by Saunders describing the suitability of tooth eruption for age assessment in young children [11, 13].

The assessment of tooth development has retained its importance in age assessment until today because tooth development is primarily genetically controlled. External factors play only a minor role in tooth development [14–18]. The prime role of genetic control applies to both tooth formation and eruption [14, 19]. The essential influence of genes explains the reproducible chronological processes of tooth development.

Although eruption is primarily genetically determined, it can be influenced by external factors. This refers in particular to mechanical obstructions of the eruption, e.g. due to lack of space or diseases of the bone such as cysts or odontomas [20]. Dentitions with such phenomena must therefore be excluded from dental age assessment. There has also long been evidence that eruption may be dependent on ethnicity [21]. In this regard, further systematic research is desirable in the future.

The third molar eruption can be assessed both clinically and radiologically. For many years, the clinical eruption of the third molars into the oral cavity was an important feature in age assessment [22–24]. However, when evaluating radiographs, it is also possible to consider conditions prior to eruption through the gingiva, which primarily means the alveolar bone above the tooth crown [25].

Various methods of staging have been presented for the evaluation of the mandibular third molar’s eruption on radiographs [13, 25–28]. These classifications differ in detail such as the reference points or in the attention to detail of their descriptions.

The intention of the present study was to compare two established as well as one newly derived simple classification for the radiological evaluation of third molar eruption. The research question of the study was to compare the correlation with age and the inter- and intra-rater reliability of the methods on the basis of a well-defined reference population.

## Material and method

The orthopantomograms (OPGs) were obtained from a university dental clinic (Münster, North Rhine-Westphalia region, Germany). For this comparison study, we sought a sample of 200 digital orthopantomograms from an equal mix of males and females in line with previous studies published in the field [29]. However, to account for later exclusions, the number of OPGs originally approached was inflated to 220. Inclusion criteria were that the quality of the images had to be sufficient in terms of the research question, as teeth 38 [FDI] and 48 had to be radiologically detectable. In addition, subjects with displaced or retained third molars were excluded. Retentions were measured according to established clinical criteria, e.g., an angle of more than 30° in the mesiodistal direction as an exclusion criterion [30, 31]. Furthermore, individuals with evidence of genetic eruption disorder or other eruption obstacles were excluded.

The dental clinic comprised different departments. Therefore, the study population was composed of patients from dental surgery, orthodontics, prosthodontics, and conservative dentistry. In addition, the images, which were all taken for medical indication, were retrospectively evaluated in terms of the present research question.

The age of the participants at the time of the X-ray examination had to be known beyond doubt. Radiographs were assessed in DICOM format at appropriate workstations using synedra Personal View software version 21.0.0.4 (synedra information technologies GmbH, Innsbruck, Austria). For the evaluations, the software’s magnification tool and the gray level adjustment tool were regularly used. The examiners were three board-certified dentists.

The evaluations were performed according to the following classifications:

*Olze *et al*.* (2012) (“Olze”) [26]

- 1. Coverage of the occlusal surface with alveolar bone
- 2. Alveolar emerge: complete resorption of the alveolar bone over the occlusal surface
- 3. At least half the crown length of the second molar has been reached, the occlusal plane has not been reached
- 4. Complete emergence in the occlusal plane
- 5. Elongation (Not listed in the original by Olze et al.)

*Willmot *et al*.* (2018) (“WHL”) [13]

- 1. Developing tooth within bone
- 2. Cusp tips at or just above alveolar bone level
- 3. Cusp tips considerably above the alveolar bone level but not fully erupted
- 4. Fully erupted

*New classification* (“New”)

- 1. Bone lamella completely intact over the occlusal surface
- 2. Alveolar emerge: bone lamella no longer completely intact, but not fully resorbed
- 3. Bone lamella completely resorbed, half the crown length of the second molar not yet reached
- 4. At least half the crown length of the second molar has been reached, the occlusal plane/occlusal surface of the second molar has not yet been reached.
- 5. Occlusal plane reached, no elongation
- 6. Elongation

Before the actual study, a calibration of the examiners took place to compensate for any differences in experience with the method. To this end, the following was performed: Prior to the main examination, 50 randomly selected images, which were not included in the main examination, were evaluated by each examiner. After this step, images were consensually determined by all three examiners together in which there was a difference of more than one stage. For the main examination, radiographs were evaluated in random order and independently of each other. One randomly selected examiner evaluated the entire study population a second time.

Data management and statistical analyses were performed in Stata, version 13.0 (Stata Corp LP, College Station, TX, USA). Tooth 38 and 48 staging was investigated as means of potentially classifying persons of unknown age into age groups. Following classification by the three raters, according to each method, the distribution of ages was subsequently compared across the stages of each method. Spearman’s rank correlation coefficient evaluated the correlation between age and stage. Age was then regressed on stage for each method and sex, adjusting for the tooth. The degree to which rating might explain the variation in age was assessed through the adjusted coefficient of determination (adj-*R*^2^) and the specific proportion of variance explained (*ω*^2^) by rating. Krippendorff’s alpha was used to evaluate the agreement between and within raters [32]. The repeatability of one rater and the reproducibility of all three raters were investigated for each method as means of evaluating the reliability of each method.

## Results

For the present study, a total of 211 out of the 220 originally approached digital OPGs from 109 females and 102 males aged 15 to 25 years could be included in the study. Table 1 shows the composition of the study population according to age and sex.

**Table 1:** Age and sex distribution of the sample

Age	Females (*n*)	Males (*n*)	Total (*n*)
15	12	12	24
16	3	11	14
17	9	11	20
18	12	10	22
19	11	9	20
20	9	10	19
21	13	7	20
22	10	10	20
23	9	9	18
24	9	7	16
25	12	6	18
**Total (*n*)**	**109**	**102**	**211**

**Fig. 1:**
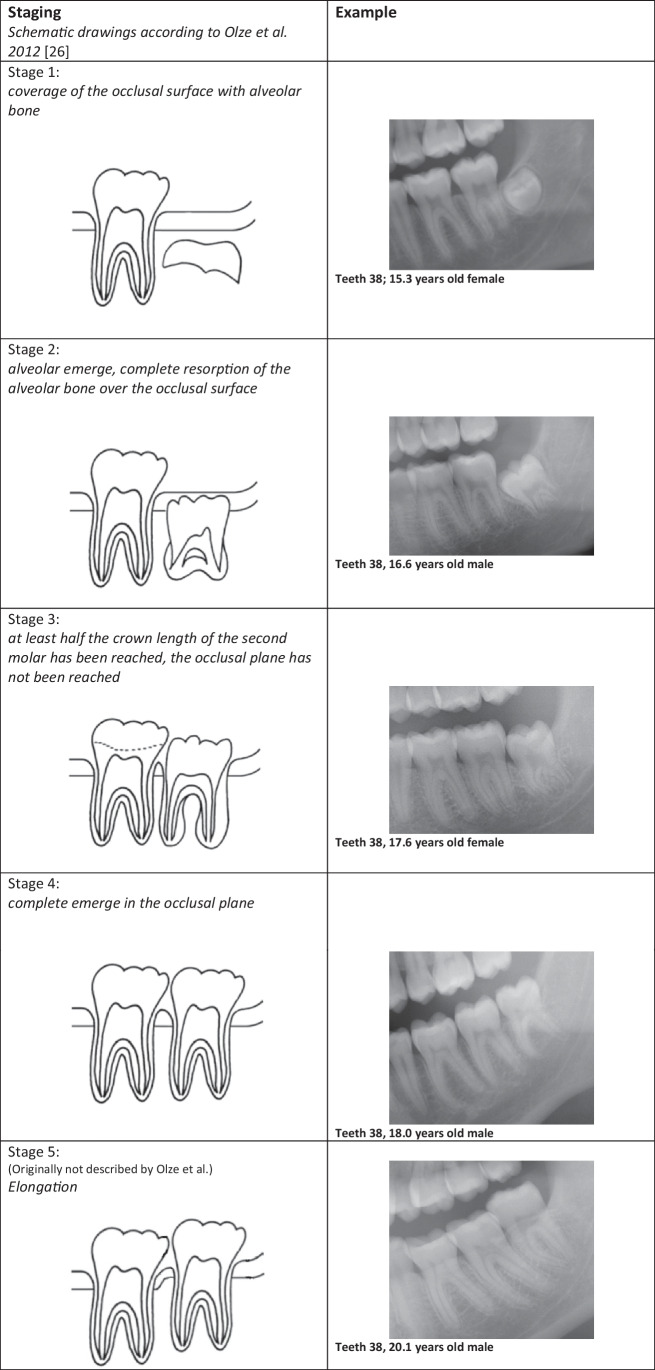
Staging according to Olze et al. (2012). Examples from the present cohort

Using any of the three methods investigated in this study, all stages of tooth development were represented. Thus, for the method according to Olze et al., all stages from 1 to 5 were detectable in the cohort, just as for the method according to Willmot et al. (1 to 4). For the newly proposed method, all stages 1 to 6 were also detectable in the examined cohort. Figure 1 shows examples of the different stages according to Olze et al. from the cohort for tooth 38.

The method of Olze et al. (Olze) and the new method (New) presented in this paper achieved a similar degree of correlation between age and stage, which were the same to two decimal places, with Spearman’s coefficients, *ρ* = 0.45 for females and *ρ* = 0.58 for males. For the Willmot et al. method (WHL), the Spearman coefficients between age and stage were *ρ* = 0.44 and *ρ* = 0.57 for females and males, respectively. There was a better correlation between age and the stages of all methods in males compared to females (Table 2), although the highest correlation was 0.583 (95% CI 0.535, 0.631) for Olze in males whereas among the females the highest was 0.446 (95% CI 0.388, 0.506) using the new method. This pattern was reproduced by the *ω*^2^ statistics, in which tooth staging from Olze et al. explained the greater proportion of variance with *ω*^2^ = 0.333 95% CI (0.281, 0.378), while among females this was the new method (*ω*^2^ = 0.247 95% CI (0.195, 0.290)). The lowest *ω*^2^ was attained by the WHL method for males (0.314 95% CI (0.261, 0.359)) and females (0.227 95% CI (0.178, 0.271)).

**Table 2:** Spearman’s correlation between stage and age, for each method and sex, with the adjusted *R*^2^ coefficient and partial omega-squared (*ω*^2^) value for the stage from the regression of age on stage for each method and sex, adjusted for tooth

Method	Sex	Spearman’s ρ (95% CI)	Adjusted *R*^2^	Partial *ω*^2^ (95% CI) for stage, adjusted for tooth
Olze	Male	0.583 (0.535, 0.631)	0.333	0.333 (0.281, 0.378)
WHL	Male	0.568 (0.515, 0.620)	0.313	0.314 (0.261, 0.359)
New	Male	0.579 (0.532, 0.627)	0.326	0.327 (0.273, 0.371)
Olze	Female	0.445 (0.384, 0.504)	0.245	0.245 (0.195, 0.289)
WHL	Female	0.440 (0.380, 0.503)	0.226	0.227 (0.178, 0.271)
New	Female	0.446 (0.388, 0.506)	0.246	0.247 (0.195, 0.290)

Intra-rater reliability was very high across all methods, ranging from 0.782 for WHL in females to 0.904 for Olze in males (Table 3). Inter-rater reliability for all methods was high ranging from 0.756 for the new method to 0.797 for Olze in males with similar results achieved, respectively, for females from 0.766 to 0.792 (Table 4).

**Table 3:** Krippendorff’s coefficients measuring intra-rater repeatability for each method by sex. *α*. Krippendorff’s alpha; *LCL*. lower confidence limits; *UCL*. upper confidence limits

Method	Sex	α	95% LCL	95% UCL
Olze	Male	0.904	0.854	0.954
WHL	Male	0.898	0.845	0.951
New	Male	0.897	0.846	0.949
Olze	Female	0.898	0.846	0.949
WHL	Female	0.782	0.712	0.853
New	Female	0.884	0.830	0.938

**Table 4:** Krippendorff’s coefficients measuring inter-rater reliability for each method by sex. *α*. Krippendorff’s alpha; *LCL*. lower confidence limits; *UCL*. upper confidence limits

Method	Sex	α	95%LCL	95%UCL
Olze	Male	0.797	0.744	0.850
WHL	Male	0.790	0.735	0.844
New	Male	0.756	0.702	0.810
Olze	Female	0.792	0.740	0.844
WHL	Female	0.774	0.719	0.828
New	Female	0.766	0.712	0.819

## Discussion

Very similar values for correlation with age and reliability were obtained for the classification systems investigated. Thus, the systems are qualitatively very close to each other. Nevertheless, the method according to Olze et al. tended to achieve the nominally best values. The similarity in performance between the staging methods reflects not only how each method categorizes eruption into comparable levels, but also how the classification systems differ only in certain details. Notably, no superior values were found for the newly developed staging system. By classifying eruption into a greater number of stages, the new method could potentially offer a greater degree of resolution, by which to classify age by stage of eruption during the years when growth is manifest. However, no advantage was apparent from the statistics measuring the correlation between age and stage, with considerable overlap between stages in the distribution of age, as observed in the other methods.

In terms of inter-rater reliability, there were no significant differences, although the method by Olze et al. marginally outperformed the others. There are no generally binding limits for a “good” agreement since the absolute values vary greatly dependent on the distribution of the levels of the rating scale [32, 33]. Krippendorff himself calls for values of at least 0.80, while values from 0.67 would only allow “tentative conclusions” [32]. However, considering that Krippendorff’s alpha is a markedly conservative measure, some authors find a value of 0.75 sufficient for a “good” agreement [34]. Following this conclusion, the values for the inter-rater agreement are in the “good” range for all methods investigated. Thus, in particular for the Olze method, reliability sufficient for practical use could be found.

The marginally superior performance of the method according to Olze et al. in this study could be in part due to its history of development, with incremental improvements over the years. The method presented by Olze et al. in 2012 is a specific enhancement of a previous method by Olze et al. from 2007 [8, 9, 26, 35]. In the previous method, a criterion for the gingival eruption was when at least one cusp tip of the erupting third molar had penetrated the gingiva [35]. While the clinical determination of this stage did not pose any problems in clinical practice, the radiographic assessment of the gingival eruption is challenging or often impossible [26]. Therefore, Olze and colleagues decided to develop a staging independent of gingival morphology, insofar as the method from 2012 has been specifically adapted to X-ray examination conditions. Although Olze et al. updated their method in 2012 for use on OPG, results are still being published using the previous iteration of the method by several author groups [36–38].

In another study, Olze et al. investigated the correlation of their stages with age. For this purpose, they evaluated 211 conventional OPGs of 109 women and 107 men aged 15 to 26 years [29]. They examined the correlation between age and eruption stage with the η-coefficient. When only the two mandibular third molars were considered, η-coefficients between 0.610 and 0.699 were obtained, depending on the observer [29]. However, caution should be exercised in ascribing a cause for the difference between our results and those of Olze, which is likely to also reflect sampling heterogeneity. While the study of Olze et al. was no doubt conducted with sufficient rigor, our study has sought to include more data with three instead of two raters. Furthermore, while the η-coefficients presented by Olze et al. indicate by how much variance in age is “explained” by tooth stage in an analysis of variance of age on stage, the omega-squared statistic presented in our results is less biased and affected by the size of the sample. Additionally, we also presented the Spearman correlation coefficient as an estimate of the correlation between age and the rank order of tooth stages [39].

The values for inter-rater agreement presented by Olze et al. in 2012, ranging from 0.874 to 0.927, were significantly higher than the values obtained in the present work. However, as discussed, these were based on only two investigators. Additionally, we presented agreement estimates using the more robust Krippendorff’s alpha measurement, although these yielded very similar values to kappa [32, 34, 40].

For the present study, a fifth “elongation” stage was added to the classification of Olze et al. from 2012. This was done because a further time span from physiological eruption into the occlusal plane to elongation can be assumed. The authors of the present study conjectured here another information about the age. However, elongation in the restricted definition does not represent a physiological state and does not occur when the dentition is appropriate in the maxilla [41]. Accordingly, stage 5 could only be detected in 2 cases in the present cohort. As a consequence, the effect of adding the additional stage remains low.

The method of Olze et al. has also been presented to be suitable for use in magnetic resonance imaging (MRI). However, this is explicitly the method from 2007 in which gingival eruption is assessed—not the modified version from 2012 [42, 43]. It must be taken into account here that the gingiva can probably be assessed much better in MRI than in OPG. It can therefore be assumed that the method with the assessment of gingival eruption will retain its relevance when used in MRI. For the OPG, the 2012 method used in this paper should continue to be used.

The Willmot et al. method evaluated in this study was presented in 2018 [13]. Comparable values were found for the method in terms of correlation with age and reliability.

Overall, the method represents a further development or simplification of Bengston’s method from 1935 [44]. The details of Bengston’s method were further modified by AlQahtani et al. and Liversidge and Molleson in 2010 and 2017, respectively [27, 45]. In particular, the authors modified the reference point of stage 3. It must be noted that the definition in the 2018 paper with the wording “considerably above the alveolar bone level” is the least defined version [13]. AlQahtani et al. in 2010 used the phrase “midway between the alveolar bone and the occlusal plane” [27]. Liversidge and Molleson in 2017 refer to “cusp tip(s) at maximum bulbosity of adjacent tooth if present” [45].

However, it is obvious that the exact determination of these reference points in the 2010 and 2017 versions caused problems in practice, which is why the new amendment was made in 2018.

All the more remarkable is the fact that Corns et al. published a study in 2021 in which the method variant from 2017 was used again [46].

In 2018, Willmot et al. presented their method originally not for use on third molars [13]. The authors applied their method in 2018 to seven left mandibular teeth with the exception of the third molars. A total of 946 OPGs from 457 girls and 489 boys aged 3 to 16 years were examined. Willmot et al. also related the eruption stage to root mineralization. The authors conclude that the method should be suitable for age assessment [13]. Ultimately, the data of Willmot et al. cannot be compared with the data of the present study because of the different study populations and the difference in research questions. However, the method was included in the present comparison because its perspective is interesting. The bone level is used as a reference point. This reference is basically well-suited for assessment in the OPG. In contrast to the method by Olze et al. (2012), it is not clearly defined to what degree the bone must be resorbed for the determination of stage 2. Furthermore, the verbalization provides a little more leeway in the assessment, as the demarcation between stages 2 and 3 is not entirely clear [13]. Due to the good inclination of the method for the OPG and the differences to the method of Olze et al., a direct comparison was required.

Overall, although the results for age prediction accuracy and reliability were good, no advantage could be found for the method compared to the method of Olze et al. (Tables 2, 3, 4).

For the present study, another new staging was proposed. The method was supposed to be even more specific for the application of the X-ray image. For this purpose, the resorption of the bone lamella over the tooth germ was further specified, and a distinction was made between partial and complete resorption. The approach was followed to further specify the method of Olze et al. (2012). The overall results for the method were inconsistent. While the method was partially superior to the Willmot et al. method for correlation with age, the new method consistently performed slightly worse for rater agreement (Table 4). It should be noted that it was the only method in the comparison that had 6 stages. All in all, contrary to expectations, no advantage could be found for the new method. Thus, the use of the classification, based on the present data, cannot be specifically recommended.

The present study faced a typical problem for studies in the field of dental age assessment: the composition of the study population. It is a problem of studies on forensic age assessment with dental radiographic methods, already discussed by Roberts and Lucas in 2021, that prospective and randomly selected study populations are not possible due to ethical restrictions with regard to radiation exposure from the X-ray examinations [47]. Thus, for such studies, X-ray images obtained on the basis of medical indications must be used. These populations are also referred to as “convenience samples” [48, 49]. Thus, in principle, the influence of the selection of the radiographs cannot be excluded. In the present study, however, this effect was not that relevant. The present study was not intended to generate reference values for the eruption of the third molars. If there was bias present, it affected all three methods studied equally.

## Conclusion

While there were no significant differences in the repeatability of the classification systems tested in terms of inter- and intra-rater agreement, the method according to Olze et al. from 2012 demonstrated marginal superiority. The method of Olze et al. from 2012 is a reliable method for future studies.

## Data Availability

The datasets analysed during the current study are available from the corresponding author on reasonable request.
